# A cell behavior screen: identification, sorting, and enrichment of cells based on motility

**DOI:** 10.1186/1471-2121-6-14

**Published:** 2005-03-22

**Authors:** Sarah L Windler-Hart, Kwan Y Chen, Anjen Chenn

**Affiliations:** 1 Department of Pathology, Feinberg School of Medicine, Northwestern University, Chicago, IL 60611, USA

## Abstract

**Background:**

Identifying and isolating cells with specific behavioral characteristics will facilitate the understanding of the molecular basis regulating these behaviors. Although many approaches exist to characterize cell motility, retrieving cells of specific motility following analysis remains challenging.

**Results:**

Cells migrating on substrates coated with fluorescent microspheres generate non-fluorescent tracks as they move and ingest the spheres. The area cleared by each cell allows for quantitation of single cell and population motility; because individual cell fluorescence is proportional to motility, cells can be sorted according to their degree of movement. Using this approach, we sorted a glioblastoma cell line into high motility and low motility populations and found stable differences in motility following sorting.

**Conclusion:**

We describe an approach to identify, sort, and enrich populations of cells possessing specific levels of motility. Unlike existing assays of cell motility, this approach enables recovery of characterized cell populations, and can enable screens to identify factors that might regulate motility differences even within clonal population of cells.

## Background

Many developing tissues are comprised of morphologically indistinguishable cells. However, these cells are often heterogeneous with respect to gene and protein expression, as well as developmental potential. Differences that develop from initially clonal cancer cell populations underlie the emergence of cells resistant to initial therapeutic intervention, and the ability of certain cancers to spread may relate in part to the intrinsic motility of cancerous cells [1]. Methods that facilitate the identification and isolation of cells exhibiting specific behaviors may lead to greater understanding of molecular mechanisms underlying cancer progression.

The identification of differences in gene and protein expression that contribute to carcinogenesis depends crucially on the specific identification and isolation of abnormal cells. Although recent advances in tissue microdissection enables highly specific isolation of cells from tissue samples [2], the ability to identify and isolate living cells based on specific behavioral characteristics may provide valuable insights that may not be evident from static morphological analysis of tissue [3,4].

Although several methods to examine cell motility exist, most characterize motility on a cell population basis, cannot distinguish heterogeneity within a population, and do not permit isolation of cells with specific motility. Variations of a classic chemotactic assay initially described by Boyden [5] have been effectively used to characterize the motility of a variety of cell populations. These assays typically monitor the movement of cells to the opposite side of a porous membrane onto which they are initially plated in high numbers. Typically, Boyden/transwell assays reveal differences in motility of the most motile fraction of the entire populations analyzed, because the vast majority of cells do not pass through the transwell membrane. Finally, although these assays have proven to be quite versatile, they require large starting numbers of cells, and isolation of cells possessing distinct motility remains challenging.

It has been observed that cells moving on substrates coated with supra-colloidal gold particles generate a record of their movements by clearing the particles from their path [6]. This clearing of a particle-free trail by a combination of cell locomotion and phagocytosis, described as "phagokinetics," has been used to quantify the motility of a variety of cell types [6-8]. Here, we describe a method that enables quantitation of motility both by direct measurement of cleared area and by fluorescent signal intensity within single cells, and permits isolation of cells based on their motility.

## Results

### Migrating cells create non-fluorescent tracks on fluorescent microsphere-coated substrates

Non-cytotoxic fluorescent polystyrene microspheres have been utilized as cell labels [9], microinjectable cell tracers [10], retrograde neuronal markers [11], and phagocytosis indicators [12]. We have taken advantage of the phagokinetic ability of migrating cells [6] by allowing them to ingest fluorescently labeled polystyrene microspheres coated onto a variety of migratory substrates. Tissue culture vessels prepared by pre-treatment with poly-D-lysine were coated with 1 μm diameter green fluorescent microspheres. Cells were plated onto polylysine-treated tissue culture plastic, incubated for 18–24 hours, and then fixed with 4% paraformaldehyde.

**Figure 1:**
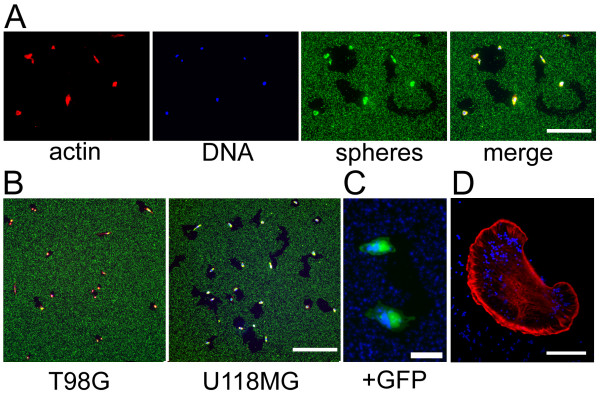
**Appearance of cells migrating on fluorescent microspheres**. (A) F-actin (red), DNA (blue), microspheres (green), and merged view indicate that cells clear non-fluorescent tracks in the dense particle field as they move. Bar, 100 μm. (B) Cell lines exhibit differences in motility reflected by the area of particles cleared, highlighted by comparing T98G and U118MG glioblastoma lines. Bar, 300 μm. (C) Cells transfected with expression vector for GFP can be visualized on a blue fluorescent microsphere field. The trails from both cells converge at a common origin, suggesting that the two cells arose from the division of a common progenitor and migrated away. Bar, 20 μm. (D) Confocal section of phalloidin-stained U118MG cell migrating on field of blue microspheres. Bar, 20 μm.

Moving cells generated microsphere-free areas in the dense fluorescent particle coat that were easily visualized using fluorescence microscopy (Figure 1). Differences in the motility of two glioblastoma cell lines were readily apparent by the distinctions in area of the tracks cleared (Figure 1B). Utilizing beads of different fluorescent emission wavelength allowed simultaneous visualization of tracks and cells stained with distinct fluorescent markers (F-actin with Alexa-Fluor 546 Phalloidin, DNA with Hoescht 33342, or transfected with green fluorescent protein, Figure 1A). Confocal imaging of cells stained with Alexa-Fluor Phalloidin migrating on microspheres reveals that ingested microspheres do not interfere with the resolution of the actin cytoskeleton (Figure 1D).

**Figure 2:**
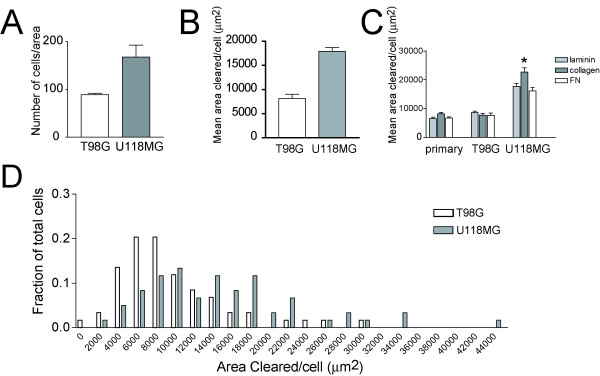
**Characteristics of fluorescent phagokinetic assay**. (A) Transwell cell motility assay. A greater number of U118MG cells (1.33 fold) transit through the filter compared with T98G. Graph depicts the mean number of cells that transit through the filter of 5 10X fields in 4 replicate wells/cell line (20 fields/cell line/experiment) from 3 independent experiments; p = 0.0185). (B) Mean area of fluorescent particles cleared per cell reveals U118MG cell line exhibits greater intrinsic motility than T98G (2.29 fold greater area cleared/cell). Graph depicts the mean motility of cells from 3 independent experiments; 100 cells/cell line measured for each experiment; p = 0.0012. (C) Fluorescent phagokinetic assay reveals differences in motility of cell lines (primary mouse cerebral cortical astrocytes, T98G, U118MG) on different extracellular matrices (fibronectin (FN), type IV collagen, laminin). * indicates p < 0.001 comparing motility of U118MG on FN vs. Collagen IV or FN vs. laminin. For all pairwise comparisons of U118MG on any substratum vs. either T98G or primary astrocytes, p < 0.001. (D) Histogram of distribution of areas cleared by U118MG vs. T98G from a representative experiment on poly-D-lysine treated tissue culture plastic (no additional substratum). Results are expressed as means +/- SEM, and statistical significance was evaluated by Student's t-test (A, B)or one-way ANOVA followed by Newman-Keuls *post-hoc *test (C).

To establish a reference for comparing the motility of two glioblastoma cell lines, U118MG and T98G, we employed a commonly used transwell filter assay. Cells were seeded on the top of the membrane and allowed to migrate to a lower compartment containing media. In contrast with traditional chemotactic transwell assays, we did not use a chemoattractant gradient, but instead utilized the normal growth media on both sides of the membrane (identical media used in fluorescent phagokinetic assay above). Thus, instead of chemotaxis, this assay measures pure cell motility. We observed that more (1.33 fold greater) U118MG cells had migrated through the transwell filter compared with the T98G cell line (Figure 2A).

To determine whether the fluorescent phagokinetic assay provided a measure of cell motility comparable to that of the transwell assay, we measured the fluorescence-free areas cleared by single cells plated on fluorescent microspheres. By tracing and measuring cleared areas generated by cells, we obtained a direct measure of how far individual cells moved in the time since plating. The area cleared per cell was obtained for at least 100 cells/cell line/independent experiment. The fluorescent phagokinetic assay showed that cells from the U118MG cell line cleared a greater mean area (2.29 fold greater) compared to the T98G cell line, an observation confirming that found with the transwell motility assay. (Figure 2B). Although these results suggest that comparable relative differences in intrinsic motility between cell lines are observed with these different assays, the phagokinetic assay has the advantage that an unbiased sample of cell motilities is measured. Unlike the transwell assay, in which only a small fraction of plated cells traverse the membrane (those with highest intrinsic motility), the motility measured with the fluorescent phagokinetic assay is more representative of the entire population of cells because the motility of all of the plated cells is recorded on the plate.

Interactions with local host microenvironment plays a crucial role in cancer spread, in part by regulating cell motility [1]. For cell migration to occur, complex interaction between cells and the extracellular matrix regulate local adhesion and cytoskeletal rearrangements [13]. To determine the utility of our approach to assess cell motility on different extracellular matrices, extracellular matrix substrates (laminin, fibronectin, type IV collagen) were coated with fluorescent microspheres, and differences in the motility of a variety of cell types on these substrates were determined (Figure 2C). To examine whether the motility of primary cells could be examined with this technique, we isolated and plated primary mouse cerebral cortical astrocytes onto the coated substrates. We observed that U118MG cells remained consistently more motile than T98G, as well as primary astrocytes on all of the three substrates tested (p < 0.001 for each pairwise comparison). The motility of U118MG cells on collagen IV was significantly greater than observed on either laminin or fibronectin (p < 0.001). These studies confirm the applicability of analyzing population motility characteristics with the fluorescent phagokinetic assay with multiple cell types and extracellular substrates.

### Fluorescence of individual cells is proportional to motility

The ability of tumor cells to acquire greater malignancy over time is well established, and despite the monoclonal origin of most tumors, they are heterogenous at clinical presentation [14]. Using the fluorescent phagokinetic assay, individual cell characteristics can be measured with fluorescent microscopy, making subtle differences in motility readily apparent. By measuring cell motility on a single cell level, we found that the motility of individual cells within clonal cell lines was distributed widely (Figure 2D and data not shown). Although documentation of individual cell motility by microscopy is straightforward, analyzing large number of cells remains cumbersome, and the desire to isolate and recover cells with differing motilities led us to consider alternatives to manual microscopic analysis.

**Figure 3:**
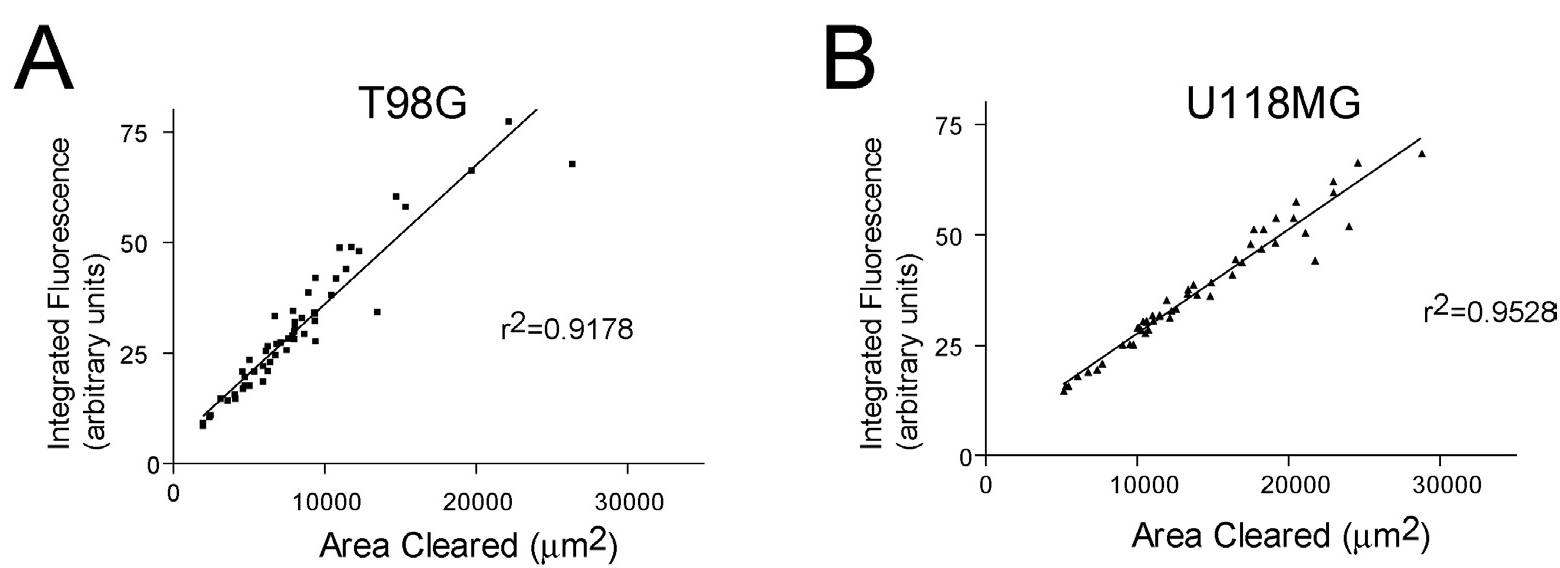
**Relationship of fluorescence and motility**. After migration on fluorescent beads, single cell fluorescence of T98G (A) and U118MG cells (B) is linearly related to area of fluorescent microspheres cleared by the cell. Fluorescence intensity of individual migrating cells was obtained by tracing each cell outline and measuring total fluorescent signal. Area cleared by each cell was measured by outlining cleared area. Images were collected using a 10X phase objective, 0.3 NA, and Endow GFP filter cube, and captured with a 16 bit CCD camera (Cascade 650, Roper Inc.).

Because the area of the tracks generated by cells relates directly to the quantity of fluorescent microspheres consumed, we reasoned that cell fluorescence could serve as another indicator of motility. By quantifying total cell fluorescence of individual cells, we found that single cell fluorescence was directly related to the area of fluorescence cleared (Figure 3 and data not shown; the differences in slope measured result from differences in gain settings between experiments to maximally utilize the dynamic range of the CCD camera). This relationship between fluorescence and motility provides an approach by which cells of different motility can be isolated by fluorescent cell sorting.

### Cells with intrinsic motility differences can be sorted and enriched by fluorescence

**Figure 4:**
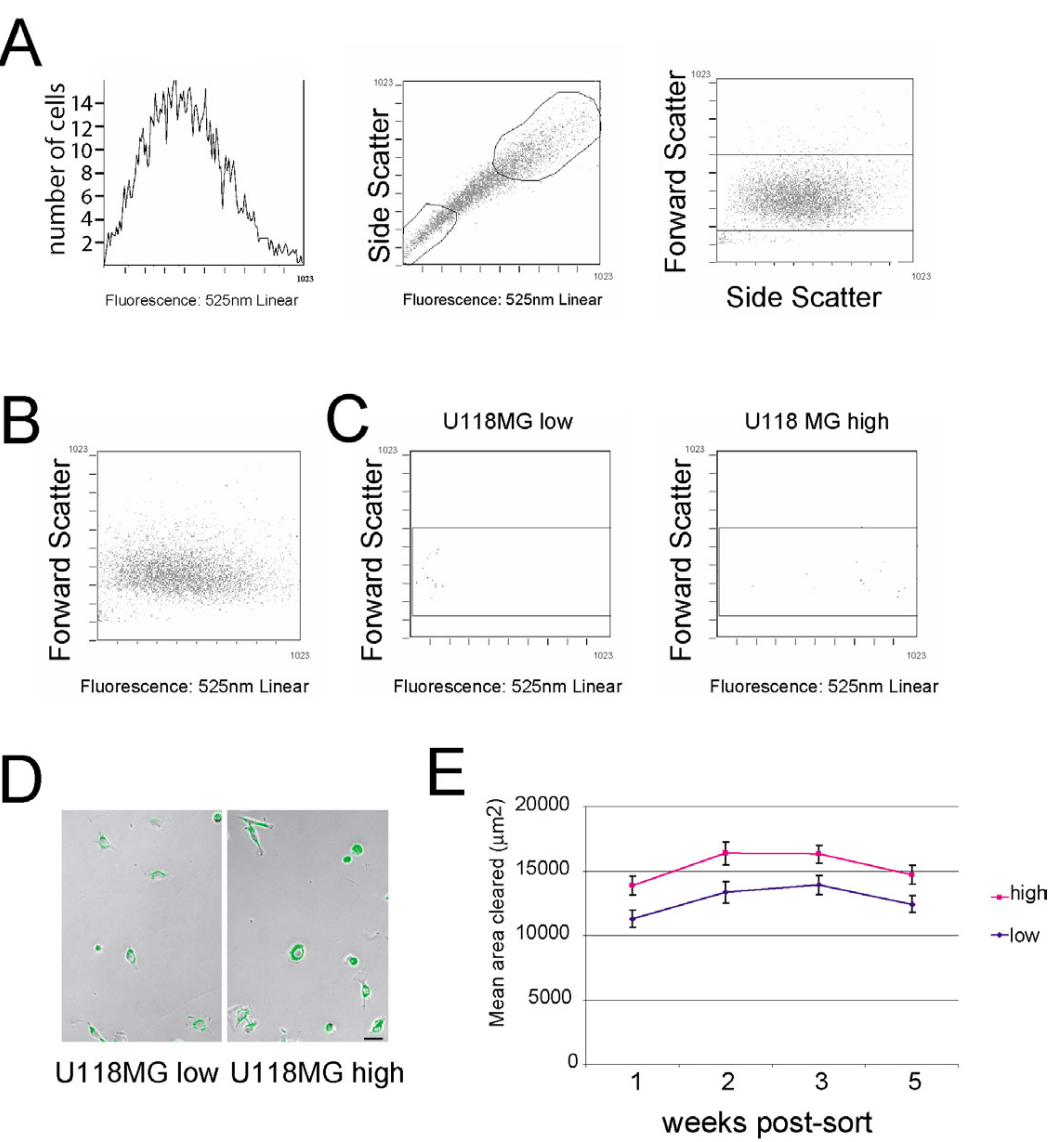
**Flow-cytometric sorting of cells based on motility**. (A) Fluorescence distribution of U118MG resembles distribution of cell motility seen in phagokinetic motility assay (left). The y-axis represents the number of cells characterized in each bin; the x-axis represents cell fluorescence at 525 nm. An untransformed linear scale for fluorescence intensity is used. On a linear scale, the voltage measured (signal intensity) is directly proportional to the channel into which the event falls. A cell with a linear value of 100 is 10 times brighter than one in with a linear value of 10. Side-scatter and fluorescence characterization of U118MG after 20 hours of migration on fluorescent microspheres (middle). Cells were sorted using the gates drawn. Granularity (side scatter) reflects the quantity of ingested beads, and is directly related to cell fluorescence. 1023 channels are available to bin the signal from fluorescence, forward, and side scatter. Forward scatter (a measure of cell size) and side scatter (a measure of cell granularity) characterization of U118MG indicates that there is no relationship between cell size and bead internalization (right). (B) Cell size is not related to cell fluorescence. Forward scatter vs. fluorescent signal of analyzed cells show no relationship between cell size and fluorescence (left). (C) Flow characterization of sorted cells. A small sample of recovered cells were re-analyzed after sorting with gates shown in (A), and both gated populations (low and high fluorescence) show no relationship between cell size (forward scatter) and cell fluorescence. (D) Images of low fluorescence cells (left) and high fluorescence cells (right) 24 hours post sorting, plated onto tissue culture plastic treated with 50 μM poly-D-lysine. Bar = 20 μm. (E) Cells retain differences in motility when reassayed after one, two, three, and five weeks post-sort (n >100 cells/cell line at each time point). Cell motility was assayed by measuring the area cleared/cell over 24 hours using the fluorescent phagokinetic assay. The overall mean fold difference between the two cell lines is 1.20, p = 0.0230. The mean motility of the high motility cell line was significantly different than the low motility cell line at each time point measured (week 1, 1.23 fold difference, p = 0.0106; week 2, 1.23 fold difference, p = 0.0105; week 3, 1.17 fold difference, p = 0.0195; week 5, 1.18 fold difference, p = 0.0201). Results are expressed as means +/- SEM, and statistical significance was evaluated by Student's t-test at each time point.

To isolate cells based on fluorescence, U118MG cells were plated on microsphere-coated culture dishes, allowed to migrate for 20 hours, and sorted (Beckman Coulter Epics Elite ESP Cell Sorter) based on fluorescence intensity and side scatter characteristics. As might be expected, fluorescence (phagocytosed spheres) correlated with side scatter [cell granularity (resulting from the microspheres)] (Figure 4A). Fluorescence intensity is not correlated with cell size (Figure 4B forward scatter (cell size) vs. fluorescence), and we have not found any morphological differences between high motility and low motility cells. After sorting the cells with gates arbitrarily chosen to select for approximately the top and bottom thirds of the total population by fluorescence, we recovered and replated high fluorescence and low fluorescence U118MG cells (Figure 4D). A small number of sorted cells were recharacterized by flow cytometry to verify the effectiveness of the sort, and as expected from the initial sort criteria used, fluorescence did not correlate with cell size [forward scatter (cell size) vs. fluorescence; Figure 4C]. When motility of post-sorted populations was determined with the fluorescent phagokinetic assay after one to five weeks in culture post-sorting, the sorted high fluorescence cells retained significantly higher motility than the low fluorescence cells (p = 0.0230; Figure 4E). These observations suggest the possibility of intrinsic and long-term heterogeneity within cell lines despite their original clonal origins.

## Discussion

The utility of using fluorescence to sort motile cells is dependent on the close relationship of bead accumulation and motility, and a number of factors may potentially confound interpretation of the described approach. Our assay is likely affected by similar factors that limit the original colloidal gold phagokinetic motility assay. Similar to the observations of multiple studies using colloidal gold particles [15], we did not observe any toxicity or ill consequences from phagocytosis of beads. Cell proliferation was unaffacted, and cells plated on microspheres could be sorted, replated, expanded, and re-examined using the same assay multiple times (data not shown). After several rounds of cell division, the beads were eventually diluted away, and the cells could be re-assayed again.

If the accumulation of beads is affected by other factors in addition to motility, fluorescence will not reflect motility accurately. We believe that bead ingestion is not affected by factors other than motility for a number of reasons: 1) the beads are adherent to the substrates and thus are not free to be ingested by cells without direct contact; 2) the tracks generated by migrating cells are continuous, bead-free paths, indicating that cells do not exhibit periods of migration without bead ingestion; 3) accumulation of fluorescent beads correlates directly with the degree of cell migration as recorded by cleared areas, as well as the quantity of beads cleared (Figure 4). Fluorescence of the cells was identical to the fluorescence of an equal area of uncleared microspheres adjacent to the cleared area (no evidence of differences by paired t-tests, data not shown), suggesting that all of the beads that are cleared are ingested by the cell.

There is undoubtedly a maximum quantity of beads that any given cell can ingest, and as the cell approaches this limit with sustained migration, the relationship of cell fluorescence and motility may diverge as fluorescence plateaus. However, in our studies, we did not observe a plateau in fluorescence after 24 hours of migration, with a linear relationship maintained between fluorescence and motility (Figure 4). It is conceivable, however, that highly motile, slowly dividing (or non-dividing) cells might approach a limit to bead consumption, or that cells may migrate differently upon the microsphere substrate than on uncoated substrates. These problems could be addressed by either using a lower concentration of beads or limiting the assay time.

Existing methods to quantify motility such as transwell assays, wound healing assays, and cell outgrowth assays can be complicated by cell division, and investigators have resorted to including mitotic inhibitors in their assays. In contrast, cells that have divided in the fluorescent phagokinetic assay are easily identified by the cleared trail leading from the two cells (example shown in Figure 1C). As a consequence, we can easily measure the path areas of single cells to exclude cells that have divided in the period since plating. Although one approach typically used to quantify motility when utilizing the phagokinetic assay is to report a total area cleared, we feel that reporting the area cleared per cell is more representative of cell motility than reporting total area cleared, as this quantity will not be confounded by cell division.

This method can complement existing assays of cell motility. It is especially useful when starting cell numbers are limiting, or when recovery of particular populations is desirable. Furthermore, in contrast to the commonly used transwell assay (which documents the movements of the highest motility cells in a population), this assay characterizes an unselected sample of the population, and can therefore provide unbiased information about motility characteristics of the entire cell population.

Behavioral readouts are the most direct screens for molecular pathways that regulate the behavior of interest. Although traditionally, we think of screens on an organism level, here we describe screening for differences in a specific cell behavior. Populations of cells can be selected by *in vivo *behaviors as well. For example, characterization of cells selected for the ability to metastasize revealed a number of genes involved with tumor cell invasion [16]. As demonstrated here, further resolution on a single cell basis can be valuable even in apparently homogeneous or clonal cell populations, as individual cells can exhibit wide variations in motility. To gain additional insight onto the molecular mechanisms underlying differences in cell behavior, reporter assays for gene expression with single cell fidelity [17], combined with genome-wide analysis of expressed genes [18,19] could be used to complement our behavioral assay in screens.

Here, we observe persistent differences in motility of a clonally-derived cell line. Further characterization can determine the molecular underpinnings that generate the differences in intrinsic cell motility. It is apparent that further heterogeneity exists within the sorted populations (data not shown); our approach enables 1) further enrichment of populations by re-sorting previously enriched cell populations and 2) finer resolution of cell populations by sorting into single cells with subsequent characterization using a variety of approaches [4,18,19].

## Conclusion

The advantages of the fluorescent phagokinetic assay relative to existing assays of cell motility are as follows. First, the preparation of substrate is straightforward, consisting merely of applying the fluorescent microspheres to the substrate, and allowing them to adhere. Second, the assay is highly sensitive- single cell characteristics, including the behavior of individual transfected cells, can be obtained in an unbiased fashion. Finally, because cell fluorescence is directly related to the area of fluorescent particles cleared, distinct subpopulations of cells can be sorted and enriched based on degree of motility. This assay could be used to identify factors that might regulate motility differences even within clonal population of cells. This approach potentially may be extended beyond two dimensions by creating three dimensional suspensions of fluorescent particles in solid matrices to also provide screens to identify additional, critical parameters of motility and oncogenesis *in vivo*.

## Methods

### Transwell migration assay

Cell migration through transwell filters was analyzed as previously described [20], with minor modifications. Briefly, 1 × 10^5 ^T98G and U118MG cells were seeded on the top of transwell membranes treated with 50 μg/ml poly-D-lysine for 30 min (8 μm pore diameter; Becton Dickinson) in media (DMEM) with 20% fetal bovine serum in both upper and lower compartments and allowed to migrate. After 6 hours, filters were fixed with 4% paraformaldehyde (15 min, 4 deg C), cells from the top surface of the filters were removed by a cotton swab, and nuclei stained with Hoescht 33342. Cells that had migrated through to the bottom surface of the filter were then counted under UV fluorescence, and confirmed by visualization with phase optics (5 fields/filter using a 10X phase objective). The average number of cells in four replicate wells was determined for each cell line in each of three independent experiments.

### Fluorescent phagokinetic migration assay

Tissue culture vessels were prepared by pre-treatment with 50 μg/ml poly-D-lysine for 30 min at room temperature (RT), then coated with 1 μm diameter fluorescent microspheres [Fluospheres (carboxylate-modified, yellow-green, Molecular Probes F8815, or carboxylate-modified, blue F8814, 0.005% in Dulbecco's phosphate buffered saline (DPBS)] for 2 hours (RT), and washed three times with DPBS. For migration on various extracellular matrices, tissue culture dishes precoated with fibronectin, collagen IV, laminin (Biocoat, BD Biosciences catalog numbers 354428, 354402, 354404) were rinsed and coated with beads as above. Cells were plated onto these substrates at a density of ~4 cells/mm^2 ^in DMEM with 20% fetal bovine serum, incubated for 18–24 hours, and then fixed with 4% paraformaldehyde, 4 deg C, 15 minutes, and washed with DPBS.

### Analysis of motility

Images of fluorescent cells on green microspheres were collected using a 10X phase objective, 0.3 NA, and Endow GFP filter cube. A 16 bit CCD camera (Cascade 650, Roper Inc.) was used to capture images. Cleared areas generated by cells were traced, and cleared area and total fluorescence of individual cells measured using the Metamorph imaging program. To prevent confounding results by overlapping paths and dividing cells, only areas cleared by single cells were measured and counted. To correlate cleared areas with fluorescence intensity, all images were collected using the same exposure time (25 ms), which was determined emperically to ensure that no pixels in the collected images were saturated. Confocal images were obtained on a Zeiss LSM510.

### Transfections and staining

Cells were transfected with expression plasmid for EGFP using Lipofectamine 2000 following manufacturer's protocols. 18 hours after transfection, cells were plated onto microsphere coated dishes and allowed to migrate for 18–24 hours. Following migration assay, cells were permeablized with 0.3% Triton X-100 in phosphate buffered saline (PBS), and actin was visualized by staining with Alexa 546- labeled phalloidin (0.15 μM in PBS for 20 minutes, room temperature; Molecular Probes); DNA was visualized by incubation with Hoescht 33342 (1 μg/ml in PBS for 5 minutes, room temperature; Molecular Probes)

### Flow cytometry

U118MG cells were plated on microsphere-coated 10 cm tissue culture dishes, incubated for 20 hours, removed using 0.25% trypsin – 1 mM EDTA, and spun at 180 g for 10 minutes. Approximately 2.6 × 10^5 ^cells were sorted (Beckman Coulter Epics Elite ESP Cell Sorter) based on fluorescence intensity and side scatter characteristics. After sorting with gates arbitrarily chosen to select for approximately the top and bottom thirds of the total population by fluorescence, 4.1 × 10^4 ^highly fluorescent U118MG's and 2.4 × 10^4 ^lower fluorescence cells were recovered and returned to culture. Fluorescent signal and forward/side scatter signal is displayed on a linear scale, using 1023 available channels of resolution. Fluorescence intensity at 525 nm is displayed as typical for the flow cytometer used; there are 1023 bins into which the signal can be resolved (12 bit analog to digital converter (ADC)). An untransformed linear scale for fluorescence intensity is used. On a linear scale, the voltage measured (signal intensity) is directly proportional to the channel into which the event falls. A cell with a linear value of 100 is 10 times brighter than one in with a linear value of 10. As typical for flow cytometry, gain and threshold is set within an experiment so that the fluorescent signal is resolved into these channels. Similarly, forward and side scatter signal height is displayed in typical fashion (binned into 1023 channels). The y-axis for figure 4A histogram represents the number of cells in each of the histogram bins.

### Primary astrocyte cell culture

Primary mouse cerebral cortical astrocytes were isolated from newborn mouse cortices as described in [21] with the following modifications. After dissection, the cells were plated on untreated tissue culture plates at 1 × 10^5^/ml in serum free DMEM with G3 glial supplement (Gibco). Cells were maintained in cultures and motility assayed within three passages.

## Authors' contributions

SH optimized the experimental protocols, participated in the design of the experiments, performed motility assays, flow sorting experiments, and assisted in drafting the manuscript. KC collected microscope images of migrating cells, quantified cleared areas, and performed the motility assay on different extracellular matrices. AC conceived of the study, participated in its design and coordination, performed the statistical analyses, quantified fluorescence vs. motility, and drafted the manuscript. All authors read and approved the final manuscript.
